# Natural Transformation as a Mechanism of Horizontal Gene Transfer in *Aliarcobacter butzleri*

**DOI:** 10.3390/pathogens10070909

**Published:** 2021-07-19

**Authors:** Marina Bonifácio, Cristiana Mateus, Ana R. Alves, Emanuel Maldonado, Ana P. Duarte, Fernanda Domingues, Mónica Oleastro, Susana Ferreira

**Affiliations:** 1 CICS-UBI-Health Sciences Research Centre, University of Beira Interior, 6201-506 Covilhã, Portugal; marina_acb@hotmail.com (M.B.); cristiana.lopes.mateus@ubi.pt (C.M.); ritalves95@gmail.com (A.R.A.); apcd@ubi.pt (A.P.D.); fcd@ubi.pt (F.D.); 2 C4-UBI-Cloud Computing Competence Centre, University of Beira Interior, 6200-284 Covilhã, Portugal; eman.maldonado@gmail.com; 3 National Reference Laboratory for Gastrointestinal Infections, Department of Infectious Diseases, National Institute of Health Dr. Ricardo Jorge, 1649-016 Lisbon, Portugal; monica.oleastro@insa.min-saude.pt

**Keywords:** *Aliarcobacter butzleri*, natural competence, transformation

## Abstract

*Aliarcobacter butzleri* is an emergent enteropathogen, showing high genetic diversity, which likely contributes to its adaptive capacity to different environments. Whether natural transformation can be a mechanism that generates genetic diversity in *A. butzleri* is still unknown. In the present study, we aimed to establish if *A. butzleri* is naturally competent for transformation and to investigate the factors influencing this process. Two different transformation procedures were tested using exogenous and isogenic DNA containing antibiotic resistance markers, and different external conditions influencing the process were evaluated. The highest number of transformable *A. butzleri* strains were obtained with the agar transformation method when compared to the biphasic system (65% versus 47%). *A. butzleri* was able to uptake isogenic chromosomal DNA at different growth phases, and the competence state was maintained from the exponential to the stationary phases. Overall, the optimal conditions for transformation with the biphasic system were the use of 1 μg of isogenic DNA and incubation at 30 °C under a microaerobic atmosphere, resulting in a transformation frequency ~8 × 10^−6^ transformants/CFU. We also observed that *A. butzleri* favored the transformation with the genetic material of its own strain/species, with the DNA incorporation process occurring promptly after the addition of genomic material. In addition, we observed that *A. butzleri* strains could exchange genetic material in co-culture assays. The presence of homologs of well-known genes involved in the competence in the *A. butzleri* genome corroborates the natural competence of this species. In conclusion, our results show that *A. butzleri* is a naturally transformable species, suggesting that horizontal gene transfer mediated by natural transformation is one of the processes contributing to its genetic diversity. In addition, natural transformation can be used as a tool for genetic studies of this species.

## 1. Introduction

The historically developed genus *Arcobacter* was proposed by Vandamme in 1991 as belonging to the class Epsilonproteobacteria and the family *Campylobacteraceae* [1]. Nonetheless, recently, a taxonomic reassessment of this genus was proposed, which was transferred to the *Arcobacteraceae* family, and it was divided into six genera, including the *Aliarcobacter* genus [2,3]. This later is a widespread and diverse genus, with the species *Aliarcobacter butzleri*, *Aliarcobacter cryaerophilus*, *Aliarcobacter skirrowii* and *Aliarcobacter thereius* being frequently associated with human and animal disease [4,5,6]. Amongst these species, *A. butzleri* is the fourth most frequently found *Campylobacter*-like-organism in human diarrheal stool samples [7,8,9] and one of the most frequently isolated bacterial pathogens in fecal samples from individuals with acute enteric disease [6]. Beyond its association with human diseases, this species has a wide distribution through the environment–animal–human web [5]. This may be associated with a high adaptive capacity, which, in turn, may be related to the high diversity of its genome, a consequence of the genomic plasticity and cellular responses [5,10]. One of the strategies for bacterial evolution is the acquisition and incorporation of foreign genetic material through horizontal gene transfer (HGT) [11]. HGT can occur by different processes, such as conjugation, transduction or natural transformation [12,13]. Natural transformation is a process characterized by the absorption, incorporation and functional expression by bacteria of extracellular DNA, which is free and often abundant in the environment and hosts [11,14]. Besides the possible benefits of natural transformation in accelerating bacterial adaptation, the acquired DNA could be used for other processes, such as nutrient supply, and genetic material for the repair of DNA damage [15]. Natural competence for transformation is recognized for more than 80 bacterial species [16], including for *Campylobacter* genus that is closely related to *Aliarcobacter*. The frequency of natural transformation for *Campylobacter* is described to reach up to 10^−3^ transformants/CFU, being the most effective form of HGT reported for this species, with a greater affinity for acquiring DNA from siblings [17,18,19,20]. *Campylobacter jejuni* is competent under ideal growth conditions, with natural transformation being influenced by environmental conditions and physiological processes [18,20].

However, little is known regarding the natural transformation ability of *A. butzleri,* even though whole-genome sequencing of *A. butzleri* has revealed several putative competence genes [10]. In the present study we aimed to characterize and advance in the understanding of the natural transformation in *A. butzleri* while contributing to improving the genetic manipulation of this bacterium. 

## 2. Results

### 2.1. Screening for Aliarcobacter butzleri Isolates with Detectable Levels of Natural Transformation

To investigate the natural competence in *A. butzleri*, we started with a screening of 17 non-related *A. butzleri* strains obtained from different sources and different genetic backgrounds [21] (Table 1). The natural transformation was carried out by two different methodologies: an agar transformation method and a biphasic system method. As donor DNA, the total genomic DNA from an *A. butzleri* mutant strain, possessing a kanamycin resistance marker inserted in the *areB* gene, coding for an inner membrane protein of an efflux pump system (DQ40A1Δ*areB::kan^R^*) was used. Amongst the 17 strains evaluated, 11 (65%) were found to be transformable with the agar transformation method and eight (47%) with the biphasic system. For the remaining six strains, no detectable levels of natural transformation were observed in the tested conditions (Table 1). In general, a higher number of transformants were obtained with the agar transformation method (a median value of 61 transformants) than with the biphasic system (a median value of 15 transformants). There were, however, two exceptions for which more than 500 transformants were obtained with the biphasic method, the DQ40A1 strain, corresponding to the native strain from the mutant used as a donor, and the CR1132 strain. In the control assays, where no donor DNA was added, no mutants were detected. In order to confirm the transformation event, the insertion of the kanamycin resistance marker was confirmed by PCR. Following this first screening, the *A. butzleri* DQ40A1 strain showed high transformation frequency and was therefore selected for additional studies, including a genomic analysis.

### 2.2. Effect of Growth Conditions in the Transformation Frequency of Aliarcobacter butzleri

In order to evaluate the factors influencing the natural transformation of *A. butzleri*, we used the DQ40A1 strain as recipient strain and biphasic system. To assess the effect of growth conditions on transformation frequency, different temperatures and atmospheric conditions were evaluated. The transformation was measured by the frequency of cells that acquired resistance to antibiotics. 

**Figure 1:**
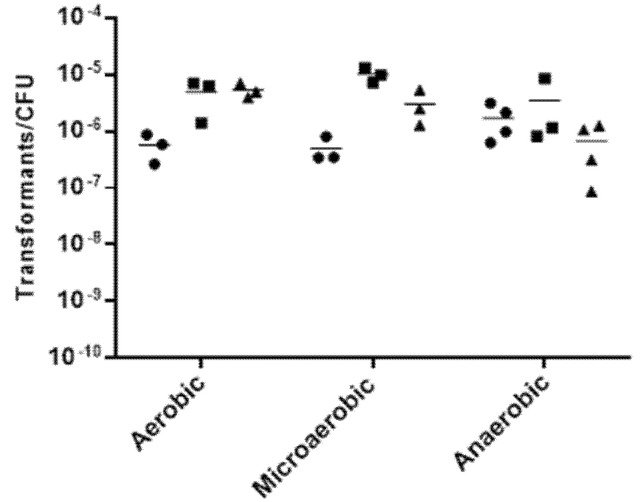
The effect of varying temperature (●20 °C; ■ 30 °C; ▲37 °C) and atmospheric conditions in transformation frequency of *Aliarcobacter butzleri* DQ40A1, using 1 µg of isogenic chromosomal DNA (DQ40A1Δ*areB*:*:kan^R^*). Results from at least three independent assays.

*A. butzleri* DQ40A1 strain was naturally transformable at all the temperatures and atmospheric conditions tested, although at varying levels (Figure 1). The highest transformation frequency was obtained with incubation at 30 °C in a microaerobic atmosphere, which was significantly higher than the obtained at 20 or 37 °C, *p* < 0.01, while at 20 °C, under aerobic and microaerobic conditions, the frequency was significantly lower (*p* < 0.05). 

**Figure 2:**
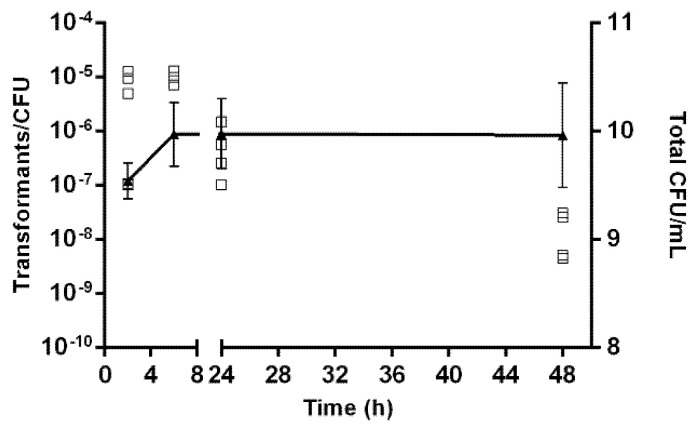
The growth curve and transformation frequencies of *Aliarcobacter butzleri* DQ40A1 strain. Growth was quantified by CFU/mL counting (▲) being presented as mean ± standard deviation, and transformation frequency was evaluated by adding isogenic chromosomal DNA (DQ40A1Δ*areB*::*kan^R^*) at different time points (□). Results from at least three independent assays.

To further explore the transformation frequency according to *A. butzleri* growth, the donor DNA was added at different time sets after *A. butzleri* inoculation. The results show that *A. butzleri* is naturally transformable at all the tested periods and different growth phases, showing competence in the exponential and stationary phases; however, with higher efficiency during the initial phase of growth, when DNA was added at 2 or 6 h after inoculation (Figure 2). In fact, the transformation frequency was significantly higher when DNA was added at 6 hours after inoculation than at 24 or 48 h of incubation (*p* < 0.05).

For the following studies, and unless otherwise stated, the assays were performed at 30 °C, under microaerobic conditions, by adding donor DNA after 6 h of bacterial inoculation.

### 2.3. Effect of DNA Concentration on Transformation Frequency of Aliarcobacter butzleri

**Figure 3:**
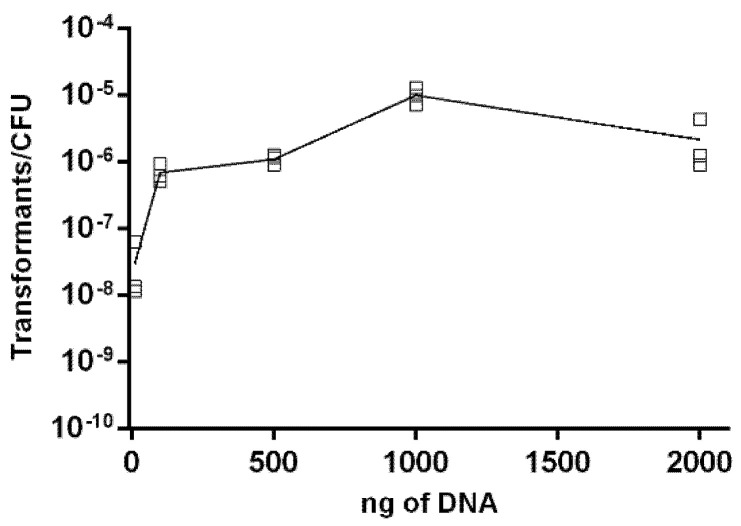
The effect of the quantity of donor DNA in the transformation frequency. The *Aliarcobacter butzleri* DQ40A1 strain was transformed with isogenic chromosomal DNA (DQ40A1Δ*areB::kan^R^*). Results are from three independent assays (□).

To evaluate whether the natural transformation was limited by the DNA concentration of the donor strain, increasing amounts of genomic DNA (gDNA) from *A. butzleri* DQ40A1Δ*areB::kan^R^* mutant were used to transform *A. butzleri* DQ40A1 strain. The experiments with different DNA concentrations showed that transformation by isogenic donor DNA resulted in saturation, after a peak in the frequency of transformation was reached with 1000 ng of donor DNA (~7.65 × 10^−6^ transformants/CFU per µg of DNA) (Figure 3). The use of 1000 ng of donor DNA led to a significantly higher frequency of transformation compared to the other tested concentrations (*p* < 0.001). The minimum quantity of DNA necessary to produce transformants was found to be 10 ng.

### 2.4. Transformation of Aliarcobacter butzleri with Different Donor DNA

We further investigated the influence of the type of transforming DNA in the transformation frequency. For that, the naturally competent *A. butzleri* DQ40A1 strain was exposed to 1 µg of different types of DNA molecules from the 344 bp PCR fragments from different strains, corresponding to a fragment of the *gyrA* gene with the single nucleotide polymorphism C254T conferring resistance to ciprofloxacin or gDNA carrying the *Kan^R^* resistance marker granting resistance to kanamycin (Table 2). We observed that the transformation frequency increased with donor DNA length and with isogenic material (Table 2). Although the transformation was possible with donor DNA from different strains, the results show that the homology between the donor DNA and the DNA of the recipient strain influences the transformation. The use of isogenic DNA is more efficient (CR1132Δ*areB::kan^R^* vs. DQ40A1Δ*areB::kan^R^*), with transformation frequency being significantly higher than that obtained with the other donor DNA (*p* < 0.0001) (Table 2). When using gDNA from *Campylobacter coli* (873 isolate) containing a gentamicin resistance marker (*aphA-3* gene) [22], no transformants were obtained (Table 2). These assays also showed that linear PCR fragments could serve as donor DNA without the need for vectors construction for transformation.

### 2.5. Kinetics of Natural Transformation

**Figure 4:**
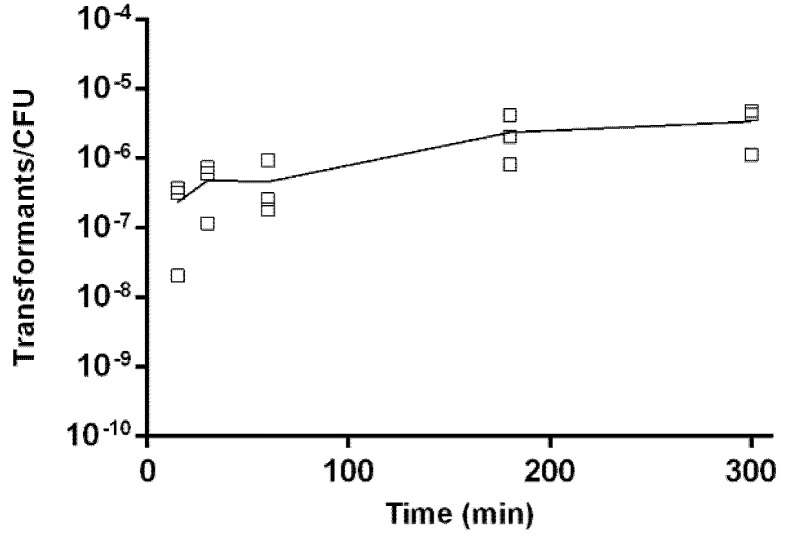
Kinetics of natural transformation. *Aliarcobacter* *butzleri* DQ40A1 strain transformed with isogenic genomic DNA (DQ40A1Δ*areB::kan^R^*). The transformation was terminated by adding DNase I at various time points. Results are from three independent assays (□).

Kinetics of natural transformation was evaluated using 1 µg of isogenic gDNA at 30 °C in microaerobic conditions with the biphasic system. The results showed that natural transformation occurred shortly after 15 min of incubation with the donor genetic material, and transformants were obtained at all the tested time points (Figure 4). A slow increase in transformation frequency was observed in the time frame from 15 min to 5 h raising from (2.4 ± 1.9) × 10^−7^ to (3.4 ± 2.0) × 10^−6^ transformants/total CFU.

### 2.6. Transfer of Antibiotic Resistance Determinants in Aliarcobacter butzleri Co-Cultures

**Figure 5:**
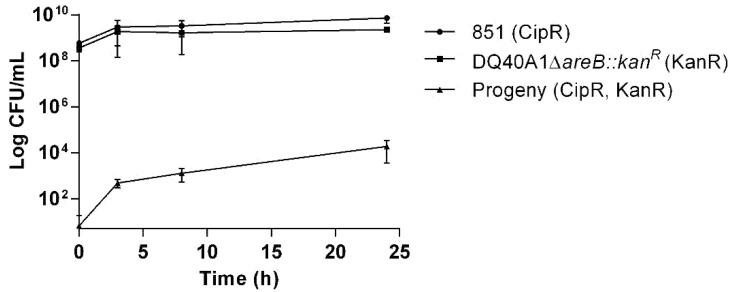
The transfer of antibiotic resistance determinants in co-cultures of Aliarcobacter butzleri carried out at 30 °C in microaerobic conditions. The progeny was evaluated by determining the number of cells resistant to both ciprofloxacin and kanamycin. Values are presented as mean ± standard deviation.

HGT has already been demonstrated in bacterial co-cultures for various bacterial species, such as *C. jejuni* [20]. In order to evaluate if transformation occurs in co-cultures of *A. butzleri*, we mixed the *A. butzleri* isogenic strain 851 carrying the C254T mutation in *gyrA* (conferring ciprofloxacin resistance) and the DQ40A1Δ*areB::kan^R^* mutant (conferring kanamycin resistance). Three hours of co-culture resulted in progenies resistant to both antibiotics, and in addition, there was an increase in the number of transformed colonies over time of culture, suggesting that the transference of genetic information proceeds during co-culture (Figure 5).

### 2.7. Identification of Putative Competence Genes in Aliarcobacter butzleri DQ40A1

To investigate whether *A. butzleri* has the genetic machinery necessary for natural transformation, we searched the genome of *A. butzleri* DQ40A1 strain (GCA_902500765.1) for homologs of competence genes of *C. jejuni*, one of the best-studied species from the *Campylobacteracea* family regarding natural transformation [17,23]. Several homologs of genes known to be implicated in natural transformation in *C. jejuni* were found in the *A. butzleri* genome (Table 3). When no homologs of *C. jejuni* were found, we examined the *A. butzleri* DNA sequences against *Neisseria gonorrhoeae* proteins. 

Overall, several homologs of genes encoding for DNA uptake, processing and other proteins related to the natural transformation process were found in the *A. butzleri* DQ40A1 genome. Considering the machinery associated with DNA uptake, it is recognized that some *cts* (for *Campylobacter* transformation system) proteins are involved in the transport of DNA for natural transformation across the outer membrane into the periplasm and over the inner membrane into the cytoplasm [17,23,24,25]. *A. butzleri* DQ40A1 harbors various homologs genes from *C. jejuni* machinery, namely a homolog of *ctsD* from *C. jejuni*, which, in turn, is a homolog to *pilQ* of *N. gonorrhoeae* encoding for the outer membrane pore for DNA transport into the periplasm [25]. In addition, homologs of *ctsG*, *pilG* and *ctsE* were also found in *A. butzleri* genome. The product of *ctsG* encodes for a putative pseudopilin-like protein in *C. jejuni*, while *pilG* is required for transformation and pilus biogenesis in *N. gonorrhoeae* [24,25], and *ctsE* encode putative nucleoside triphosphatases or nucleoside triphosphate binding protein [24]. Homologs of *ctsX* and *ctsP* seem to be absent in the *A. butzleri* genome. The absence of homologs for *ctsP* was also noticed for non-*C. jejuni* species, such as Campylobacter lari [17]. In contrast, a homolog of *comEC*, encoding for a predicted integral membrane channel for transport of DNA into the cytoplasm and considered essential for natural transformation in *C. jejuni* [26], can be found in *A. butzleri* genome. Moreover, a homolog of *comE* was found in the *A. butzleri* genome, whose product acts as a periplasmic DNA receptor contributing to the natural transformation of *C. jejuni*, however, is not considered essential for the process [27]. A homolog of PriA, shown to be involved in neisserial DNA transformation [28] was also found. Furthermore, *A. butzleri* carry genes coding for DNA processing machinery, such as the case of the homolog of the DNA processing A protein (DprA), described as having a role in DNA translocation, and which while being essential for transformation by chromosomal DNA in *Haemophilus influenza* [29], is not essential in *Helicobacter pylori* or *C. jejuni* [30,31]. Finally, homologs of other genes that may be associated with natural transformation in *C. jejuni*, although with a less established role [23], were found, namely *ctsW*, *proC* and *ceuB*, but with less than 50% of homology.

**Table 3:** Competence protein homologs in the *Aliarcobacter butzleri* DQ40A1 strain.

*Campylobacter jejuni*			*Aliarcobacter butzleri* RM4018 ^a^	*Aliarcobacter butzleri* DQ40A1 (CDS)		
Category and Homolog (Locus ID)	Gene Product	Length (AA)	Homolog (Locus ID)	Homolog (Locus ID)	Length (AA)	Sequence ID (%) [DNA (AA)] ^b^
**DNA Uptake**						
*ctsD* (cj1474c)	Component of type II secretion/type IV pilus system (potential outer membrane pore)	472	ABU_RS09160	GDI89_RS08280	478	46.8 (20.9)
*ctsP* (cj1473c)	Component of type II secretion/type IV pilus system (putative NTPases/NTP binding protein)	202	NA	NA	NA	NA
*ctsX* (cj1472c)	Component of type II secretion/type IV pilus system	195	NA	NA	NA	NA
*ctsE* (cj1471c)	Component of type II secretion/type IV pilus system (putative NTPases/NTP binding protein)	519	ABU_RS08225	GDI89_RS05100	453	53.5 (36.1)
*pilG* ^c^	Competence pseudopilus	410	ABU_RS08220	GDI89_RS05105	396	38.4 (19.9)
*ctsG* (cj1343c)	Putative periplasmic protein	171	ABU_RS03485	GDI89_RS04080	132	47.5 (28.1)
*comEC* (cj1211)	Membrane transporter protein involved in the transfer of DNA across the membrane	419	ABU_RS06285	GDI89_RS02780	409	56.6 (36.2)
*comE* (cj0011c)	Periplasmic DNA-binding competence protein	79	ABU_RS11390	GDI89_RS07505	123	43.7 (31.7)
*priA ^c^*	Helicase	729	ABU_RS06960	GDI89_RS02485	599 ^d^	36.6 (26.6)
**DNA Processing**						
*dprA* (cj0634)	DNA processing protein	257	ABU_RS01180	GDI89_RS06255	258	57.3 (41.3)
*recA*	DNA recombination protein	343	ABU_RS11180	GDI89_RS02220	349	73.5 (73.4)
**Others**						
*ctsT* (cj1077)	Putative periplasmic protein	100	NA	NA	NA	NA
*ctsW* (cj1028c)	Purine/pyrimidine phosphoribosyltransferase	191	ABU_RS02380	GDI89_RS03920	190	59.2 (44.0)
*ctsR* (cj1475c)	Hypothetical protein	105	NA	NA	NA	NA
*proC* (cj1076)	Putative PCA reductase	243	ABU_RS02955	NZ_CABVRU010000127.1 + NZ_CABVRU010000230.1 ^e^	254	53.1 (40.5)
*ceuB* (cj1352)	Enterochelin uptake permease	322	ABU_RS05530	GDI89_RS07350	320	49.1 (25.4)

^a^ Reference Assembly RM4018 column provides accession determined by NCBI blastn (i.e., by searching the corresponding DQ40A1 CDS). ^b^ Sequence %ID was determined with needle software from EMBOSS package (v.6.6.0.) [32] for both DNA and amino acid (AA) data types by aligning the DQ40A1 CDS with the original sequence indicated in (*Campylobacter jejuni* column). ^c^ Homology with *Neisseria gonorrhoeae* proteins of NZ_CP012028.1 genome. ^d^ The identified sequence is a partial CDS (truncated). ^e^ Sequence assembled from two partial CDS obtained from described locus ID. NA—Not Available.

## 3. Discussion

Natural transformation is a well-known mechanism of HGT that allows bacteria to acquire new genetic traits, resulting in genetic diversity among a population and contributing to adaptation to new environments and hosts [12,25]. For this process to occur, bacteria require to be naturally competent, having the ability to capture macromolecules of DNA from the environment and transfer them into a new cell where they will recombine with genomic DNA and replicate [16,25,33]. Several bacterial species have been described as naturally competent for transformation [16]; however, this has not yet been studied in *A. butzleri*. Furthermore, the genetic manipulation of *A. butzleri* has remained remarkably uncharacterized, with a small number of studies using genetic modification approaches.

The high genetic diversity of *A. butzleri* is likely associated with its natural competence for DNA uptake, which in turn can facilitate recombination between strains. To evaluate whether *A. butzleri* is naturally transformable, 17 strains were tested with two natural transformation methodologies. We found that 65% of the strains studied were naturally transformable with the agar method, e but only 47% using the biphasic procedure. Differences in the transformation capacity between strains of the same species have been reported for several bacterial species, including *C. jejuni*, *C. coli* [19,34], *Haemophilus influenza* [35], *Gallibacterium anatis* [36] or *Actinobacillus actinomycetemcomitans* [37]. In the present study, most of the isolates that showed detectable levels of natural transformation were from animal origin, more specifically from poultry. Kim et al. (2006) also found a dependence of the transformation capacity with the origin of the *C. coli* strain, with turkey-origin strains presenting higher transformation frequencies than swine-origin strains, likely due to differences in their genetic background or due to specific transformation requirements [34]. Another possible cause that can explain these results is the eventual presence of certain mechanisms, such as the secretion of endonucleases, that can limit the transformation in particular environments [13,16,38]. 

The natural transformation process is considered to occur under normal growth conditions; however, it can suffer the influence of external factors [12,16] that can play an important role in transformation efficiency and frequency. Therefore, besides the genetic background of the strains, factors such as the growth phase, incubation conditions, and the nature, type and size of the donor genetic material are also relevant [13,18,39,40]. 

Considering the temperature and atmosphere composition, these are two of the conditions impacting the process of natural transformation for several bacteria, such as *C. jejuni* [18]. Temperature had a greater influence in the frequency of *A. butzleri* transformation when compared to the atmospheric conditions, with a reduction of temperature resulting in a decrease in the number of transformants, under both aerobic and microaerobic conditions. Although *A. butzleri* was naturally transformable at all the temperatures and atmospheric conditions studied, even in limiting growth conditions, the most favorable conditions for the transformation of *A. butzleri* were established at 30 °C in a microaerobic atmosphere. Accordingly, for *C. jejuni*, transformation was favored when carried out under the optimal growth conditions for these bacteria. Furthermore, for *C. jejuni*, the temperature also had a greater influence on the transformation frequency, when compared to the atmospheric conditions [18].

The results showed that *A. butzleri* is naturally competent for transformation during all the growth phases, with a maximum frequency of transformants being obtained when the genetic material was added at 2 or 6 h after bacterial inoculation. This profile was similar to that observed for other related bacteria, such as *C. jejuni*, *C. coli* or *H. pylori*, with the natural transformation occurring at all the growth phases as well, although for these species, the frequency of transformation reaches a peak at the mid-exponential phase [18,19,41]. However, the regulatory pathways of natural transformation are divergent according to bacterial species/genera [13,42], and the most efficient process can occur at different growth phases, either when DNA is added prior to the exponential phase [41], during the exponential phase [18,19,43,44] or during the late-log to mid-stationary phase [42].

In the environment, bacteria are kept in contact with diverse concentrations of genetic material that may influence the frequency of natural transformation. In *A. butzleri*, the number of transformants was directly correlated with the concentration of the donor genetic material, reaching a maximum with the use of 1 μg of gDNA. This amount of DNA was also described for other Gram-negative bacteria, such as *C. jejuni*, *C. coli* and *G. anatis*, as being necessary to reach the highest number of transformants [19,36], while other species require much less, such as *H. pylori*, for which 6 ng of gDNA was described [41]. 

The size and homology of the donor DNA can also have an impact on natural transformation since homologous recombination is required for efficient incorporation of the uptaked DNA. Regarding size, the decrease in fragment size is associated with a decrease in the region available for homologous recombination between the donor and the recipient DNA, resulting in a reduction of transformation efficiency [45]. This was observed in our study as well since when using isogenic DNA as donor genetic material, a lower number of transformants was obtained with a PCR fragment than with gDNA. This occurs despite the higher number of copies of the selective marker present in the PCR fragments when compared to the number of copies present in gDNA with the same concentration. These results may indicate that the greater the extent of homology between the incorporated sequences and the bacterial recipient genome, the greater the probability of hybridizing with the bacterial genome and, consequently, obtaining a more efficient transformation. 

The nature of the donor DNA was also evaluated, and when donor DNA from *C. coli* was used, no transformants were obtained, similarly to what happens for *C. jejuni*, for which the addition of increasing concentrations of *A. butzleri* gDNA did not originate transformants [18]. This negative result is likely associated with insufficient lack of homology between the gDNA of these species and with the presence of defense mechanisms, such as restriction-modification systems [11]. Overall, the results suggest that HGT via natural transformation between different species is less probable than within a species. 

The transformation kinetics assays showed that *A. butzleri* uptake of DNA from the environment is a fast process, as described for other bacteria, such as *C. jejuni*, *C. coli* or *A. calcoaceticus* [18,19,46]. In addition, a gradual increase in the number of transformants over time was observed, mimicking the process that occurs in *Campylobacter* spp. [18,19].

HGT also contributes to the transmission and spread of resistance determinants among bacterial communities [47]. For example, *C. jejuni* is capable of transferring resistance determinants in co-cultures without the addition of genetic material in the culture medium [20]. This phenomenon was also observed in the present study, with the exchange of chromosomally encoded antibiotic resistance determinants during a co-culture of different *A. butzleri* strains. The increase in the number of transformants observed during the co-culture can result from the constant release of DNA into the medium through cell lysis, similar to what happens in *C. jejuni* [20,26]. Thus, it is very likely that natural transformation plays a role in the transfer of genetic material, including antimicrobial resistance, among *A. butzleri* strains.

Regarding transformation machinery, the previous analysis of the *A. butzleri* genome showed the presence of putative competence genes [10]. Moreover, it was previously suggested that homologs of the *comEC* gene, encoding for a periplasmic DNA receptor contributing to the natural transformation [26], are present in the core genome of naturally transformable Gram-negative species [48]. In the present study, a homolog of the *comEC* gene was found in the *A. butzleri* genome, sharing 57% similarity with the homolog gene in *C. jejuni*. Other homologs genes encoding for proteins essential for DNA uptake and processing were also identified in the *A. butzleri* genome, corroborating the natural competence for the transformation of this bacterium. Nonetheless, machinery required for natural transformation may also be encoded by divergent homologs or unrelated proteins with the same function, a subject that needs to be further explored. 

In summary, the present study demonstrated, for the first time, that *A. butzleri* is a naturally transformable bacterium and brought knowledge regarding the natural transformation process, pointing out this process as a way of HGT within this species. A protocol to improve the molecular research of this microorganism was also established, providing a new tool for genetic manipulation of this bacterium. Nonetheless, despite the advances in our understanding of the natural transformation of *A. butzleri*, more studies are needed to unveil the mechanisms involved in the process and towards improving knowledge about the contribution of the natural transformation process to the diversity and genetic adaptation of *A. butzleri*. 

## 4. Materials and Methods

### 4.1. Bacterial Strains and Growth Conditions

The *A. butzleri* recipient strains used in this work, as well as their origin and characteristics, are shown in Table 1. Two of the donor strains, DQ40A1Δ*areB::kan^R^* and CR1132∆*areB::kan^R^*, were generated by the insertion of a kanamycin resistance cassette (*aphA-3*) interrupting the *areB* gene (ABU_RS11090). The *aphA-3*_cassette was obtained by BamHI and KpnI double digestion of the pUC18-K2 plasmid, followed by binding to the upstream and downstream region of the *areB* gene by overlap-extension PCR. The purified PCR fragment was used for mutant construction by the agar transformation method, as described elsewhere [49]. The *A. butzleri* 851 *gyrA* mutant strain was generated by transformation of the DQ40A1 strain with a 344 bp PCR fragment of the *gyrA* gene, carrying the single nucleotide polymorphism (C254T) conferring resistance to ciprofloxacin. 

*A. butzleri* strains were routinely cultured on Blood Agar Base (Oxoid, Basingstoke, England) supplemented with 5% defibrinated horse blood (*v*/*v*) (BA—Blood Agar). The bacteria were incubated at 30 °C in a controlled atmosphere (6% O_2_, ±7.1% CO_2_ and 3.6% H_2_) generated by an atmosphere modifier (Anoxomat AN2CTS, Mart Microbiology B.V., Drachten, Netherlands), unless otherwise stated. For transformants selection, strains were transferred to BA plates containing 4 μg/mL of ciprofloxacin, or 30 and 50 μg/mL of kanamycin, or both antibiotics.

### 4.2. Genetic Material Preparation

Different genetic material was used for the transformation assays: gDNA of *A. butzleri* Ab_2811, DQ40A1Δ*areB::kan^R^* and CR1132Δ*areB::kan^R^* strains, and purified PCR fragments with different sizes resulting from amplification of both the *gyrA* gene of *A. butzleri* Ab_2811 strain and the *areB* gene of DQ40A1Δ*areB::kan^R^* strain. Genomic DNA from *C. coli* 873 was also used. The GF-1 Nucleic Acid Extraction Kit (Vivantis, Shah Alam, Malaysia) was used for gDNA extraction. Primers specified in Table 4 were used for PCR amplification. The genetic material obtained was quantified using a nanoespectrophotometer, and its integrity and purity were analyzed through an agarose gel.

### 4.3. Natural Transformation Using the Agar Transformation Method

Seventeen *A. butzleri* strains were tested using a natural transformation protocol in a solid medium. Initially, each of the strains was cultured on BA plates and incubated for 24 h at 30 °C under microaerobic atmospheric conditions. After incubation, the cells were resuspended in 200 μL of Tryptic Soy Broth (TSB, Merck, Darmstadt, Germany) and spread on BA plates, incubated for a further 4 h under the same conditions. In two distinct areas of the plates, 1 µg of gDNA from *A. butzleri* DQ40A1Δ*areB::kan^R^* strain was added in a volume of 40 µL, and plates were incubated for 8 h under the above conditions. Negative controls were performed, replacing gDNA with water. Subsequently, the cultures were transferred to another BA plate and incubated for 18 h. After this incubation period, the biomass was transferred to PBS and applied to BA plates supplemented with 50 μg/mL of kanamycin. These plates were incubated for 3–7 days. The assays were performed on three independent days. The occurrence of natural transformation was confirmed for a few clones in each transformation by PCR using the primers areB_A1 and areB_B2, followed by fragment size assessment through gel electrophoresis. 

### 4.4. Natural Transformation Using a Biphasic System 

The natural transformation was also performed using a biphasic system, based on the protocol described for *Campylobacter* by Wang & Taylor (1990), with modifications [19]. Briefly, after growth on BA plates for 24 h, the *A. butzleri* DQ40A1 strain at approximately 5 × 10^8^ CFU/mL in 200 µL of TSB was transferred to test tubes containing 1.5 mL of BA and incubated for 6 h at 30 °C under microaerobic conditions. Subsequently, 1 μg of genetic material, or 40 μL of water in the control, was added to the tubes and incubated again for a further 5 h. Next, the cells were harvested and diluted in phosphate-buffered saline (PBS), and appropriated dilutions were transferred onto selective and non-selective BA plates. After incubation for 5 days, colonies were counted, and the transformation frequency was calculated. This assay was performed on three independent days. The transformation was verified by PCR for selected clones. This protocol was also used for screening natural transformation in the 17 isolates of *A. butzleri* under study, using 1 µg of gDNA from the donor *A. butzleri* DQ40A1Δ*areB::kan^R^* strain.

### 4.5. Effect of Growth Conditions

The influence of temperature and atmospheric conditions or growth phase on the transformation frequency of *A. butzleri* was determined using the biphasic system. For that, temperatures of 20, 30 and 37 °C, and aerobic, microaerobic and anaerobic conditions, were tested. Regarding the growth phase, *A. butzleri* DQ40A1 cells were incubated for 2, 6, 24 and 48 h before the addition of gDNA. After the incubation periods, a count of the total CFU/mL was performed, and then the gDNA of *A. butzleri* DQ40A1Δ*areB::kan^R^* strain was added as described above. The transformation frequencies were calculated and compared. The assays were performed at least three independent times.

### 4.6. Kinetics of Natural Transformation

To evaluate the kinetics of natural transformation in *A. butzleri* DQ40A1 strain, the biphasic protocol was used, adding to each tube 1 μg of gDNA from the *A. butzleri* DQ40A1Δ*areB::kan^R^* strain carrying the resistance kanamycin marker, and incubation at 30 °C under microaerobic conditions. Each individual transformation assay was terminated at times 0, 15, 30, 60, 180 and 300 min by the addition of 100 μg/mL DNase, after which a period of incubation of 2 h was followed, under the previously described conditions, for phenotypic expression [18]. Finally, the transformation frequency was determined, transferring 100 μL of the culture or dilutions to selective plates and for non-selective BA plates. This assay was performed three independent times.

### 4.7. Influence of Genetic Material on Natural Transformation

A saturation curve was performed at 30 °C and a microaerobic atmosphere, using the biphasic system protocol, with various concentrations of exogenous gDNA from the donor *A. butzleri* DQ40A1Δ*areB::kan^R^* strain. The following different quantities of gDNA, 0.01, 0.1, 0.5, 1 and 2 μg, were added to the culture, using *A. butzleri* DQ40A1 as a receptor strain. Furthermore, the influence of different types of genetic material was tested using amplified PCR fragments of *A. butzleri* Ab_2811 and DQ40A1Δ*areB::kan^R^* strains, as well as the gDNA previously extracted of *A. butzleri* CR1132Δ*areB::kan^R^*, DQ40A1Δ*areB::kan^R^*, Ab_2811 and *C. coli* 873 strains. Each assay was performed on three independent days.

### 4.8. Exchange of Genetic Material Between Bacterial Co-Cultures

For co-culture experiments, the strains of *A. butzleri* 851 (ciprofloxacin-resistant) and DQ40A1Δ*areB::kan^R^* (kanamycin-resistant) were collected from BA plates after 24 h of incubation and settled to grow together in the biphasic system at 30 °C and microaerobic atmosphere. From each culture, 5 × 10^8^ CFU/mL in 200 μL of TSB were added to the test tubes containing 1.5 mL of BA medium, and independent tubes were prepared for each incubation time (0, 3, 8 and 24 h). At each time, a sample was taken, and successive dilutions were carried out in PBS. Successive dilutions were then transferred to BA plates, supplemented with 30 μg/mL kanamycin or 4 μg/mL ciprofloxacin, to count the CFU/mL of each strain, or to BA plates supplemented with both antibiotics, to count the CFU/mL of the double resistant bacteria. Assays were performed on three independent days.

### 4.9. Bioinformatics Analyses—Identification of Homologs Genes Presence/Absence

The predicted CDS of competence genes from the bacterial genomes *of Campylobacter jejuni* and *Neisseria gonorrhoeae*, were downloaded from NCBI Genbank. The genome assembly of the *A. butzleri* DQ40A1 strain was locally formatted using NCBI BLAST tools [52]. The CDS set (queries) from each species were translated (translation table = 11) and searched against the genome using tBLASTn (v.2.9.0) [53] (option -db_gencode 11). The results were screened by employing an E-value threshold of 1e-4. The contigs identified in this procedure were then retrieved in full from the genome and compiled as a separate library which was used as input for Prodigal (V2.6.3) [54] software (-p meta option) to identify and retrieve all suitable CDS, by matching through the identified tBLASTn best hits.

The NCBI nr database using blastx webservice limited to *A. butzleri* (taxid 28197) was employed to ensure that the identified CDS were homologous and presented similar functions to the queries.

## Figures and Tables

**Table 1:** *Aliarcobacter butzleri* strains and transformation frequencies.

Recipient *A. butzleri* Strain	Origin/Year of Isolation	ST ^a^	Resistance Profile ^a^	Agar Transformation Method	Biphasic System Method
1426_2003	Diarrheic human stool/2003	47	NAL, CTA ^b^	+ + -	- - -
Ab_1711	Poultry slaughterhouse equipment surface/2011	ST_new1	LEV, CIP, NAL, CTA	- - -	- - -
Ab_2211	Slaughterhouse surface/2011	460	AMP, NAL, CTA	- - -	- - -
Ab_2811	Poultry carcass neck skin/2011	107	AMP, ERY, LEV, CIP, NAL, CTA	- + +	+ - -
Ab_4211	Poultry carcass drippings/2011	ST_new2	AMP, LEV, CIP, NAL, CTA	+ + +	- + +
Ab_4511	Poultry carcass drippings/2011	510	AMP, NAL, CTA	+ + -	+ + +
CR424	Poultry meat/2015	ST_new3	AMP, ERY, NAL, CTA	- - -	- - -
CR502	Poultry meat/2015	ST_new4	ERY, LEV, CIP, NAL, CTA	- - -	- - -
CR604	Beef meat/2015	ST_new5	NAL, CTA	+ + +	+ + +
CR641	Poultry meat/2015	108	ERY, NAL, CTA	+ - -	- - -
CR891	Poultry meat/2016	94	NAL, CTA	- - -	- - -
CR892	Poultry meat/2016	ST_new6	AMP, LEV, CIP, NAL, CTA	+ + +	- + -
CR1132	Ready-to-eat vegetables/2016	ST_new7	NAL, CTA	+ + +	+ + +
CR1143	Poultry meat/2016	ST_new8	AMP, LEV, CIP, NAL, CTA	+ + +	+ - +
DQ20dA1	Goat milk/2015	ST_new9	AMP, LEV, CIP, NAL, CTA	- - -	- - -
DQ31A1	Sheep milk/2015	172	AMP, NAL, CTA	+ + +	- - -
DQ40A1	Dairy plant equipment surface/2015	ST_new10	NAL, CTA	+ + +	+ + +

NT—non-transformable at the tested conditions; +—considering three independent replicates, the strain was transformed once; ++—twice, +++—thrice. ^a^ Data from reference [21]. ^b^ Borderline minimum inhibitory concentration to ampicillin. AMP—ampicillin, ERY—erythromycin, LEV—levofloxacin, CIP—ciprofloxacin, NAL—nalidixic acid, CTA—cefotaxime. In the tested conditions, the mutation frequency for the strains was <10^−9^.

**Table 2:** Frequency of transformation of *Aliarcobacter butzleri* DQ40A1 strain using homologous and heterologous PCR fragments and genomic DNA.

Donor DNA (Nature of DNA/Strain)	Resistance Marker	Length of the PCR Fragment	Transformants/CFU
gDNA/*Campylobacter coli* 873	*aphA-3*	-	NT
*gyrA* PCR fragment/Ab_2811 strain	C254T in *gyrA* gene	344 bp	(6.70 ± 2.72) × 10^−9^
*gyrA* PCR fragment/Ab_2811 strain	C254T in *gyrA* gene	1410 pb	(7.85 ± 1.13) × 10^−8^
gDNA/CR1132*ΔareB::kan^R^*	*aphA-3* (*Kan^R^* cassette from pUC18-K2)	-	(2.59 ± 0.51) × 10^−7^
PCR fragment/of DQ40A1*ΔareB::kan^R^*	*aphA-3* (*Kan^R^* cassette from pUC18-K2), flanked by upstream and downstream regions of about 400 bp	1638 bp	(3.96 ± 3.54) × 10^−7^
gDNA/Ab_2811 strain	C254T in *gyrA* gene	-	(6.92 ± 7.67) × 10^−7^
gDNA/DQ40A1*ΔareB::kan^R^*	*aphA-3* (*Kan^R^* cassette from pUC18-K2)	-	(7.65 ± 2.25) × 10^−6^

NT—no transformants.

**Table 4:** The primers used in PCR in this study.

Primers	Sequence	Size of Amplified Fragment (bp)	Reference
gyrA_Fw	TGGACGTGCATTACCAGATG	1410	[50]
gyrA_Rv	GCAACTTTTCCTTTTCCACCT		
F-QRDR	TGGATTAAAGCCAGTTCATAGAAG	344	[51]
R2-QRDR	TCATMGWATCATCATAATTTGGWAC		
areB_A1	TTGAAATAAGGGCTACTTACTCAGG	1638	[49]
areB_B2	GTTCGCTCTGGCTTGCAAAT		

## Data Availability

Data are contained within the text.
